# Influence of Imidazole-Dipeptides on Cognitive Status and Preservation in Elders: A Narrative Review

**DOI:** 10.3390/nu13020397

**Published:** 2021-01-27

**Authors:** Nobutaka Masuoka, Chenxu Lei, Haowei Li, Tatsuhiro Hisatsune

**Affiliations:** Department of Integrated Biosciences, Graduate School of Frontier Sciences, The University of Tokyo, Kashiwa 277-8562, Japan; 2201788429@edu.k.u-tokyo.ac.jp (N.M.); 7598321927@edu.k.u-tokyo.ac.jp (C.L.); 3153157958@edu.k.u-tokyo.ac.jp (H.L.)

**Keywords:** cognitive function, memory function, aging, anserine, carnosine, mild cognitive impairment (MCI), Alzheimer’s Disease (AD)

## Abstract

The worldwide increase in the number of patients with dementia is becoming a growing problem, while Alzheimer’s disease (AD), a primary neurodegenerative disorder, accounts for more than 70% of all dementia cases. Research on the prevention or reduction of AD occurrence through food ingredients has been widely conducted. In particular, histidine-containing dipeptides, also known as imidazole dipeptides derived from meat, have received much attention. Imidazole dipeptides are abundant in meats such as poultry, fish, and pork. As evidenced by data from recent human intervention trials conducted worldwide, daily supplementation of carnosine and anserine, which are both imidazole dipeptides, can improve memory loss in the elderly and reduce the risk of developing AD. This article also summarizes the latest researches on the biochemical properties of imidazole dipeptides and their effects on animal models associated with age-related cognitive decline. In this review, we focus on the results of human intervention studies using supplements of poultry-derived imidazole dipeptides, including anserine and carnosine, affecting the preservation of cognitive function in the elderly, and discuss how imidazole dipeptides act in the brain to prevent age-related cognitive decline and the onset of dementia.

## 1. Introduction

Alzheimer’s disease (AD), the most common form of dementia, is known to develop in the elderly after a period of mild cognitive impairment (MCI) [1,2,3]. According to the latest clinical diagnostic criteria, MCI persons have subjective complaints of forgetfulness, and informants who know the person well (such as family members living together) have concerns about memory deterioration [3,4]. In this stage, general cognitive function is normal, and no dementia can be observed. Within five years, about half of people with MCI will develop dementia, but most will remain in MCI. Very few of them may revert to a cognitively normal state [5,6,7,8]. When progressing from the MCI to dementia, there is a significant decline in two or more cognitive functions, including orientation and decision-making domains. Since memory dysfunction may be a precursor to the onset of dementia, improving memory dysfunction in the elderly may help reduce the risk of developing dementia [5,9,10].

Recent studies have collected plenty of knowledge about the risk of developing dementia and found that having lifestyle-related diseases (diabetes, hyperlipidemia, hypertension, and arteriosclerosis) increases the incidence of dementia [11,12,13]. In addition, the elderly present in a state of activity and muscle weakness defined as frailty, and those in the frail state are not only likely to require long-term care but are also at increased risk of dementia [14,15]. However, even if they become frail once, it is possible to return to a healthy state again with proper nutritional management [16,17,18]. In summary, dementia can be thought of as a disease that occurs in the brain as a result of a disorder of the entire body, rather than a deterioration of health limited to the brain.

Imidazole dipeptide is a dipeptide mainly contained in vertebrate muscle tissue [19,20]. It is also found in some invertebrate tissue [21]. The most common imidazole dipeptides include carnosine, anserine, balerine/ophidine, homocarnosine, acetyl-carnosine, and carnitine. Carnosine is present in human skeletal muscle and can be also detected in the brain and other tissue rather than muscle tissue, but its concentration in blood is extremely low due to the influence of carnosine degrading enzyme present in plasma in humans. In livestock meat, beef, and pork contain only carnosine, and chicken contains anserine and carnosine in a ratio of 2~3: 1. Balerine/ophidine is found in snake and whale muscle.

Recent researches have shown imidazole dipeptide exerts many important biological activities. Taking carnosine, for example, has the ability of antioxidant, anti-inflammatory, antiaggregant, and neuroprotection [20]. Carnosine thus has been regarded to play a positive role in Alzheimer’s disease (AD). At the same time, the presence of human serum carnosinase in blood brings limitations to carnosine therapy for AD. As a methylated carnosine analog, anserine is found to be more resistant to carnosinase. The combined usage of anserine and carnosine is thus thought to be a good strategy. To date, multiple double-blind, placebo-controlled, randomized controlled trials have been conducted with chicken-derived imidazole dipeptide foods (anserine/carnosine = 2~3) [16,22,23,24,25,26] and have shown beneficial effects of imidazole dipeptides on human cognitive function in the elderly. Treatment with imidazole dipeptide, carnosine or anserine, has also been shown to have positive effects on mice models of AD [27,28,29]. In this review, we discussed the effects of imidazole dipeptides, carnosine and anserine, on the preservation of brain function in humans, as well as model animals.

## 2. Methodology

We analyzed the studies related to the last 20 years (2000–2020) on the relationship between imidazole dipeptides and suppression of AD-related cognitive decline. Search PubMed/MEDLINE for a series of keywords, including imidazole dipeptides, carnosine, anserine, balenine, carcinine, acetyl-carnosine, and homocarnosine, and their effects on the protection of cognitive declines (“AD”, “Mild cognitive impairment”, “Cognition disorders”, “Cognitive decline”, “Neurodegeneration”). We analyzed the titles and abstracts of the retrieved studies to select relevant articles. Moreover, we conducted a manual search of references to relevant articles, including older human studies and animal studies, to find additional publications that might have missed through electronic searches only. The last update of our dataset was performed in December 2020.

## 3. Biochemical Properties of Imidazole Dipeptides

Since the 1950s, there have been many studies on the biochemical properties of imidazole dipeptides [30], and recent reviews have summarized their effect on brain aging comprehensively [19,20]. The effects of imidazole dipeptides on the brain are largely due to their biochemical properties, which can mainly be divided into five parts: (a) buffering activity, (b) metal ion chelating activity, (c) antioxidant activity, (d) inhibiting glycoxidation and lipoxidation, and (e) anti-aggregate activity. In addition to these five basic biochemical characteristics, a specific receptor for imidazole dipeptides has also been suggested by Nagai and colleagues that these dipeptides may act on histamine-receptors [31]. Therefore, the most distinctive feature of imidazole dipeptides is that they have imidazole rings in their molecules. Imidazole dipeptide has three isoelectric points, and the imidazole ring gives the imidazole dipeptide the buffering activity. The second pKa within a neutral pH range with a pK of 6.83 for carnosine and around 7.04 for anserine [29,32,33]. Although intracellular pH is maintained near neutral, it is known that the pH tends to become acidic due to inflammatory cellular responses. Among imidazole dipeptides, anserine, which has an isoelectric point near neutral, is assumed to have a strong effect in maintaining intracellular pH, which may help to preserve the function of brain microcapillaries in an AD model mouse [29].

Imidazole dipeptides are known to have the capability to form complexes with metals of the first transition metal series such as copper ion, cobalt ion, nickel ion, and zinc ion, which is thought to be due to their characteristic intramolecular structures. It has been widely known that imidazole dipeptides have strong chelating activity against copper and zinc [19,20,30]. Transition metal ions such as copper ions, and zinc ions are toxic in AD [34]. Studies have shown that these metal ions are related to the formation of amyloid plaques [34,35]. Both copper and zinc ions can inhibit the activity of an insulin-degrading enzyme, which is the main enzyme in Aβ degradation [36]. The metal iron chelating ability of imidazole dipeptides indicates that they may have the capability to alleviate the toxicity of metal ions in AD and may have the effect of preventing Aβ aggregation, suggested in an AD model mouse study [27].

Numerous studies have reported the antioxidant activity of imidazole dipeptides. Boldyrev et al. summarized the antioxidant mechanism of imidazole dipeptides, involving different mechanisms including metal ion chelation and reactive oxygen species (ROS) and free radical scavenging [19,37]. The negative effects of ROS on AD have been widely accepted [38]. Studies have shown that the generated ROS favors oxidative stress and neuronal death through Fenton and Haber-Weiss reactions. [39]. The metal chelating ability of imidazole dipeptide has been shown previously. In addition to this, imidazole dipeptide also can reduce the intracellular level of superoxide anions and hydroxyl free radicals and is an effective quencher of ROS [40]. The antioxidant activity of imidazole dipeptides is based on their radical scavenging activity. Kohen et al. have well demonstrated that the peroxyl radical-trapping ability of imidazole dipeptides increases in the order anserine > homocarnosine > carnosine [41]. Imidazole dipeptides can exert antioxidant effects by increasing the endogenous antioxidant protection system. It is reported that carnosine supplementation at a dose of 0.5% in food pellets (approximately 400 mg/kg/day in a rat) for 6 weeks in rats increased the activity of enzymatic antioxidants such as Superoxide dismutase (SOD) [42]. Imidazole dipeptide is also a highly effective protective agent against hypochlorite (HOCl) [43,44]. Pattison et al. found that compounds with imidazole and other free amine sites can form imidazole chloramine with hypochlorite, thereby limiting the oxidation ability of hypochlorite [43]. Imidazole dipeptides thus are considered to have a positive effect on Myeloperoxidase (MPO) related tissue inflammation, which would result in the production of hypochlorite radicals [43,44,45]. Very recently, it has been also proposed that anti-oxidant activity against hypochlorite is a candidate for the mechanism of action in the preservation of cognitive function by imidazole dipeptide supplementation in elderly volunteers [46].

Besides, it has been reported that imidazole dipeptides have inhibitory activity against advanced glycoxidation end products (AGEs) [19,47,48] and advanced lipoxidation end products (ALEs) [19,48,49]. These compounds are both related to Alzheimer’s disease. For against advanced glycoxidation end products (AGEs), imidazole dipeptides can inhibit protein glycation and reverse glycated protein through a translocation mechanism. For advanced lipoxidation end products (ALEs), several papers have reported this ability is related to a direct reactive carbonyl species (RCS) quenching mechanism [49].

All these biochemical characteristics of the imidazole dipeptide are closely linked to the inhibition of inflammatory responses. Both the antioxidant activity and the metal ion chelating activity are linked to the effect of the imidazole dipeptide for the protection of neuroinflammatory reactions, which is one of the main causes for cognitive decline in AD. Kawahara and colleagues have indicated that the protective effect of imidazole dipeptide against ischemic injuries occurred not only in the brain but also in the tissues outside the brain [50]. Studies involving glial P2Y1 receptor antagonists in both vascular dementia and AD mice models [51,52] suggest that the mechanisms by which imidazole dipeptides counteract neuroinflammation in vascular dementia caused by ischemic injury are similar to those acting against AD. The suppression of neuroinflammatory reactions may also be beneficial for inhibiting the accumulation of amyloid-β (Aβ) peptides.

The aggregation and deposition of Aβ are one of the main characteristics of Alzheimer’s disease. As early as 1995, carnosine was found to have the ability of anti-protein-cross-linking [53]. Subsequently, Hobart et al. proved that the anti-protein-cross-linking properties of carnosine depend on the imidazole functional group, suggesting the antiaggregant ability of imidazole dipeptide [54]. The inhibitory effect of imidazole dipeptide on Aβ aggregation has been confirmed both in vitro [55] and in Alzheimer’s mice model [27].

## 4. Imidazole Dipeptide on Brain Function

In considering the effects of imidazole dipeptides on the brain, it is important to discuss the concentration of imidazole dipeptides in the brain and how they can reach or act on the brain when ingested externally, both in animal models and in humans. It has been confirmed in animal models (rodents) that carnosine and homocarnosine are present in significant concentrations in the brain [19], and the presence of homocarnosine (0.5–1 mM) has been confirmed in humans by a non-invasive method using MRS [56]. The concentration of carnosine is relatively lower than that of homocarnosine in the rat brain [57], and its presence has not been confirmed by non-invasive methods using MRS. The presence of carnosine in brain tissue has been confirmed by immunohistochemical methods, suggesting its presence in glial cells rather than neurons [58]. To date, there are no data to suggest that other imidazole dipeptides, including anserine, are present in brain tissue, both in animal models and humans. The possible functions of carnosine and homocarnosine in the brain have been postulated to include antioxidant effects and modulatory effects on neurotransmission, but many are unclear.

The possible role of imidazole dipeptide in the brain is thought to be similar to that in skeletal muscle. Carnosine can reduce the accumulation of lactic acid in active muscles and prevent intramuscular acidification [59], thus forming the concept of lactic acid shuttle intracellular and intercellular [60]. The metabolic behavior of carnosine is thought to occur in glial cells. It has been reported that in astrocytes, carnosine plays a role in promoting the output of lactic acid from cells and provides metabolic support for neurons and axons [20].

Besides, the antioxidant activity of carnosine and homocarnosine is believed to have anti-inflammatory and neuroprotective functions [19]. As a metal ion chelator, carnosine can inhibit the neurotoxicity mediated by zinc and copper ions. At the same time, a study has shown that carnosine can reduce the concentrations of ROS in rat cerebellar cells [61], implying the antioxidant function of carnosine in the brain. Another neuroprotective action is that carnosine can inhibit the neurotoxicity induced by NMDA [62]. The anti-inflammatory function of imidazole dipeptides has also been confirmed in human and mice models [23,24,28,29], which we would discuss in detail in the next section. At present, the function of Imidazole dipeptide in the brain is still not very clear and needs more detailed researches.

Although, no human data are yet available to show that orally ingested imidazole dipeptide reliably reaches the brain; in animal models, studies using brain samples taken after administration have shown that ingestion increases the concentration in the brain [63]. Imidazole dipeptide can be transported by the transporters PEPT1, PEPT2, PHT1, and PHT2. PEPT1 is not expressed in either human or mouse brain. In humans, astrocytes express PEPT2, and microglia mainly express PEPT2 and PHT1 [64,65]. It is possible that orally ingested imidazole dipeptide is absorbed by the intestine and enters the blood. It may enter cells in the cerebral blood vessels or glial cells by the transporters such as PEPT2 and affect brain function. Imidazole dipeptides are originally very abundant in muscle cells. In addition, they are also present in cardiomyocytes and vascular smooth muscle [19]. Many experimental results have indicated that carnosine lowers blood pressure, and this effect may be mediated by histamine receptors, maybe an effect on NO, or may contribute directly to the contraction of vascular smooth muscle cells [31]. However, there is still no unified view as to which mechanism is responsible. Brain tissue has a very high energy requirement, and capillaries run everywhere to supply energy, including oxygen. Capillaries in the brain are thin, only about 5 microns in diameter, and are composed of vascular endothelial cells and pericytes (a type of vascular smooth muscle cell) [29]. In addition, imidazole dipeptides have been suggested to have a protective effect against diabetic kidney disease [66] and retinal degeneration [67] by acting possibly on capillaries in other body tissues. It is possible that imidazole dipeptide is taken up by these cells, including brain capillary pericytes, and acts on them [29]. The specific mechanism of oral imidazole dipeptides affecting brain function is still unclear, and whether exogenous imidazole dipeptides can be absorbed and utilized by brain cells still needs further research.

### 4.1. Imidazole Dipeptide in Animal Models of AD

To date, more than 10 species of AD model mice have been developed to search for drugs as well as nutraceuticals that are effective in preventing or progressing AD [68,69,70]. Three animal studies have been conducted to determine the effect of imidazole dipeptide on improving cognitive function using mouse models incorporating AD risk genes (Table 1) [27,28,29]. Previous studies have shown [71,72,73,74] that senile plaques accumulate at 6 months of age and memory function starts to declines. To fasten the period of cognitive decline in Alzheimer’s model mice, high-fat diet administration has been utilized to induce AD model with diabetes mellitus [28,74]. In this diabetic AD model, a high-fat diet was started at 4 months of age, and carnosine administration (1 mg/mL in drinking water, approximately 5 mg/head/day, and 200 mg/kg/day) was started 2 weeks later [28]. The effect of carnosine on the prevention of AD was examined by assessing memory behavior at 6 months of age. At this timing, a contextual fear-conditioned spatial learning test was performed to evaluate the spatial memory ability of mice. On the first day of the test, the mice were placed in a conditioning box and then subjected to electric shock stimulation to remind them of the relationship between space and fear. On the second day of the test, how much fear was recollected just by putting it in the box was quantitatively evaluated by the time ratio of the freezing response, and used as an index of the spatial memory ability of the mouse. It was found that carnosine had an effect of avoiding deterioration of memory function. After the behavior test, we found that carnosine had antioxidant and anti-inflammatory effects on the neural tissues of the hippocampal formation, but did not detect any difference in the aggregation of amyloid-β.

Corona et al. have evaluated the results of carnosine on memory function and senile plaque accumulation in a different AD model mouse (3× Tg) treated with carnosine (2 mg/mL in drinking water, approximately 10 mg/head/day) for approximately 12 months [27]. It was shown that long-term administration of carnosine from one month of age to 12 months of age suppressed the aggregation of amyloid-β, but no effect was found on tau pathology, which is another character of Alzheimer’s disease [75]. Meanwhile, only a trend toward improvement was observed. The difference between the two experiments is the difference in the length of time carnosine was administered. Corona et al. administered carnosine for a long period, from 1 month to 12 months after birth, before the beginning of the aggregation of amyloid-β before the accumulation of senile plaques began, and found an effect on the aggregation and formation of amyloid-β, whereas Herculano et al. administered carnosine for a short period, from 4 months to 6 weeks, after the aggregation of amyloid-β had already begun. On the other hand, Herculano et al. administered carnosine for a short period and found no effect on the accumulation of senile plaques, but only a protective effect against inflammation of brain tissue and cerebral blood vessels. In the study by Corona et al., they revealed the chelating effect of carnosine on divalent cations in glial cells and on the mitochondrial electron transport system in hippocampal cells [27]. On the other hand, in the study of Herculano et al., it has been observed that the effect of carnosine on cerebrovascular oxidative stress disorder and inflammatory reaction of brain tissue [28]. A diffuse inflammatory reaction accompanied by activation of microglia was also observed in the brain tissue. It was speculated that these inflammatory reactions triggered a decrease in neuronal function and a decrease in memory function. The differences in obtained results between the two similar animal model experiments may reflect the multifaceted aspects of carnosine’s action.

Carnosine is rapidly converted to beta-alanine and histidine by degrading enzymes in the blood of a human. While in rodents, in which the enzyme is absent [21], the levels of carnosinase in the blood can be maintained, and thus the administration of carnosine has better effects on AD model mice. Considering the limits of carnosine, another imidazole dipeptide, anserine, which is a methylated carnosine analog, is resistant to carnosinase to a certain extent [19], so it is less likely to be degraded and can remain in the bloodstream to some extent after ingestion. If imidazole dipeptides need to maintain their dipeptide properties to work in humans, then there is a possibility that anserine will be more effective. Therefore, Kaneko et al. conducted a study to determine the efficacy of a newly purified form of anserine from fish meat on APP/PS1 AD model mice [29]. In their study, the animals were used as 18-month cases where cognitive function in the model mice was sufficiently reduced, followed by the administration of anserine for two months. They tested whether the administration of anserine could reverse this dysfunction in animals with accumulated senile plaque and cognitive decline. A water maze test was used as a behavioral test. It was shown that the cognitive function of the AD model mice was restored by the administration of anserine. In these mice, vascular inflammation in the hippocampus and cerebral cortex, inflammatory responses in neural tissues, and elevated levels of inflammatory cytokines were found to be improved. However, similar to our previous results of carnosine, we have not been able to find any evidence of anserine inhibiting the accumulation of amyloid-β. After sufficient accumulation of senile plaques, short-term administration of anserine for 2 months did not have an effect to erase senile plaques, but the inflammatory response seen in the brain was remitted and cognitive function was restored. They demonstrated that anserine treatment (10 mg/mouse/day) recovered cognitive decline in aged AD-model mice, probably through the suppression of neurovascular-unit dysfunction and neuroinflammatory reactions that occurred in aged AD model mice. Accelerated neurovascular-unit dysfunction including brain capillary pericyte degeneration is known to cause blood–brain barrier breakdown in the brain of Alzheimer model mice and AD patients [76,77,78] and blood–brain barrier breakdown has been proved to be an early biomarker of human cognitive dysfunction [79]. Considering the resistance of anserine to carnosinase and its protective function on the neurovascular-unit, we think that anserine may be a better strategy for the treatment of people with Alzheimer’s disease than carnosine.

Currently, imidazole dipeptide has been studied mostly at the cellular level, while few studies have been conducted on mouse models of Alzheimer’s disease. Although cellular studies have shown that carnosine has an inhibitory effect on amyloid-β aggregation [55] and inflammatory cytokine release [80], more studies on AD model mice are needed to better model the effects of imidazole dipeptide on AD.

### 4.2. Imidazole Dipeptide in Human Interventional Trial Studies

In order to confirm the effect of imidazole dipeptide in humans, a total of seven human studies have been conducted to examine the effects of imidazole dipeptide on cognitive function in the elderly. These include one in Europe, two in the United States, and four in Asia (Table 2). The most common human study was a mixture of imidazole dipeptides extracted from chicken meat (1 g per day) with an anserine/carnosine ratio of 2~3. Szcześniak conducted a 3-month intervention study with residents of a home for the elderly [16]. There was an improvement in motor function and a trend toward improvement in cognitive function in the overall participants. In addition, a sub-analysis of the elderly over the age of 80 showed an improvement in cognitive function [16].

Using a similar formula containing imidazole dipeptide extracted from chicken, our research group also conducted a human intervention study in healthy elderly people. The first study (pilot study) was conducted in 2013, with participants in their 40s to 80s, totaling 69 people. The results of this study have been published across the three papers [22,23,24] Memory function tests and MRI image acquisitions with functional MRI (fMRI) [22] and arterial spin labeling (ASL) [23] protocols were performed before and after the test food to confirm the effect. In this human study, a randomized controlled trial RCT (Randomized Controlled Trial) was used in which volunteers (subjects), test food distributors, and measurers were not notified of the type of test food. Healthy volunteers were divided into two groups, one group containing 1 g of chicken imidazole dipeptide (Active group; containing anserine and carnosine 3: 1). The other group ingested a placebo granular food that resembled the shape and flavor but did not contain chicken imidazole dipeptide every day for a certain period.

The second trial (long-term study) started in 2014, with an overall duration of 12 months, and tests were conducted before, 3, 6, and 12 months after the start of the study to verify the effects of the food. The results of this study have been published in the two papers [23,25]. To examine the effect of food, subjects were tested before and after ingestion and the test data were compared between the two groups. The cognitive functions of volunteers were evaluated by the three neuropsychological tests, Wechsler memory scale-logical memory (WMS-LM), MMSE (Mini-Mental State Examination), and ADAS (Alzheimer’s Disease Assessment Scale). As described in the previous report [23], in the first RCT, the food intake period was 3 months, and data from 39 elderly people (60–80 years old) participated were obtained. In the second RCT, data from 84 elderly people (60–80 years old) after the food intake period of 6 months were obtained and analyzed. In both studies, imidazole dipeptide was shown to improve memory function (WMS-LM delayed recall) with a statistically significant difference (*p* < 0.05). In addition, the suppression of inflammatory cytokine production by this anserine/carnosine supplementation has been also reported [23,24].

Besides, brain MRI imaging examined the state of cerebral blood flow and brain function, including changes in brain blood flow by arterial spin labeling (ASL) MRI. From the results of MRI cerebral blood flow analysis, it was confirmed that imidazole dipeptide intake improved cerebral blood flow, especially in the prefrontal region [25]. Ingestion of chicken imidazole dipeptide (750 mg of anserine and 250 mg of carnosine per day) resulted in significant differences in both memory function tests and brain MRI imaging between the group that received chicken imidazole dipeptide and the placebo group.

**Figure 1:**
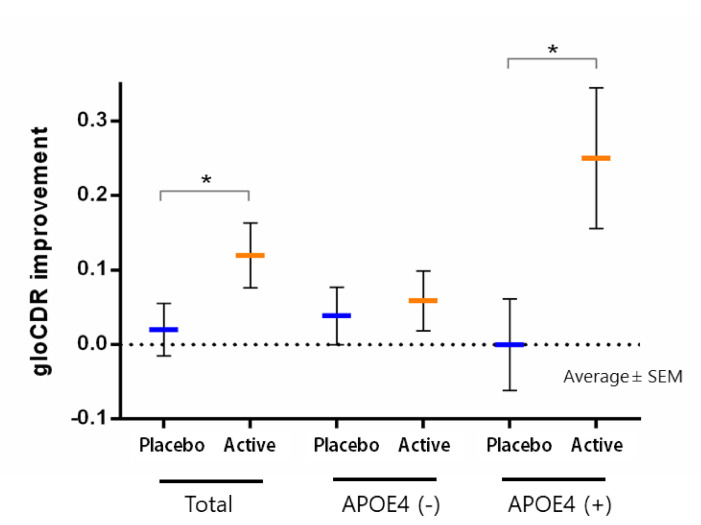
The improvement of gloCDR score in ApoE4 + mild cognitive impairment (MCI) patients, who took imidazole dipeptide supplementation for 12 weeks [26]. Average ± SEM are shown in histogram. * *p* < 0.05.

Since it was shown that 1 g/day of imidazole dipeptide had a positive effect on brain function in healthy elderly people, a similar study was conducted in mild cognitive impairment subjects. This study was conducted in collaboration with a city memory clinic [26]. Elderly people who had already been clinically diagnosed with MCI were asked to take imidazole dipeptide (750 mg of anserine and 250 mg of carnosine) supplementation (ACS) for 12 weeks, before and after which cognitive function, blood tests, and electroencephalography were performed. Cognitive function was assessed including the CDR (Cognitive Dementia Rating) test. In this study, 20 of the 50 subjects who completed the tests were ApoE4 carriers. The improvement in cognitive function was found to be stronger in the ApoE4 carriers, which are risk genes (Figure 1). The improvement of the CDR test score from 0.5 to 0 is often regarded as a sign of the reversion from MCI to cognitive normal [6,7]. The CDR test is a psychometric test commonly used in the assessment of MCI and dementia. A score of 0 on the global CDR test score closely corresponds to cognitive normality, 0.5 to MCI, and 1 to mild dementia [1,82]. In this sense, imidazole dipeptide supplementation may induce MCI reversion [26], which relates to a reduction of dementia risk [7].

In addition to a human study using a chicken-derived imidazole dipeptide mixture, an intervention study was conducted in the United States using a supplement containing carnosine with another functional ingredient [81,83]. In a study with 100 mg of carnosine, a modest effect on cognitive function was shown [81]. Besides, one intervention trial has been conducted for young to middle healthy volunteers (18–45 years old) in Thailand [84] using soup supplements extracted from chicken (the essence of chicken), which include anserine (300 mg) and carnosine (100 mg). It has been reported to have an enhancing effect on cognitive speed.

The human studies conducted to date have included imidazole dipeptide mixtures derived from chicken meat or carnosine alone, and no human studies have been conducted to verify the effects of anserine alone. This is probably because no human food product that can be used for human studies has been developed so far. Therefore, our research group established a method to highly purify anserine from fish that contain only anserine in their muscles, and manufactured test foods using the anserine purified by this method, and conducted human trials [46]. In this study, people with mild cognitive impairment were recruited in a city health promotion center. As a result, we have found that the 12-weeks single administration of imidazole dipeptide, anserine, for MCI elderly individuals have a beneficial effect on cognitive function as assessed by the MMSE test. The results of the multiple regression analysis showed that the effect was stronger for those who had a low daily intake of anserine. The effect of anserine supplementation was also stronger for those with higher daily carnosine intake [46]. Taken together with these observations, it can be noted that imidazole dipeptides of anserine and carnosine have some beneficial effects on cognitive function in elderly individuals.

### 4.3. Suggested Mechanism between Imidazole Dipeptide and Cognitive Reserve

When examining the effects of imidazole dipeptides on the brain, possible mechanisms of action include regulation of neural tissue metabolism, pH buffering, anti-inflammation, regulation of final glycation product activity, limiting the neurotoxicity of zinc and copper ions, modulation of astrocyte activity, effects on serotonin neurons, and a wide variety of mechanistic assumptions are possible at this stage, including altered behavior of noradrenaline and dopamine [85]. However, there are two main possibilities: (1): outside of brain hypothesis whether these dipeptides are acting within the blood vessels or cerebral vasculature rather than within the brain tissue, or (2): inside of brain hypothesis whether they have a point of action after reaching the brain tissue.

Concerning the possibility of (1): outside of brain hypothesis), the current view is stemmed from the affection of blood cells for the microglia found around senile plaques, or amyloid-β plaques, at autopsy in AD. The importance of innate immunity has been increasingly recognized in AD pathology, and evidence for the involvement of the inflammasome in the pathogenesis of neuro-organizing diseases is now accumulating [86,87]. It is presumed that innate immune responses within blood vessels have adverse effects on brain tissue, and possibly that anserine had some ameliorating effects on these innate immune responses within blood vessels.

It is unclear whether the 3-months of imidazole dipeptide intake was sufficient for the improvement of cognitive function. The improvement would have been stronger if the subjects had taken the medication for a longer period. Indeed, in a previous study of a 12-month imidazole dipeptide mixture, long-term treatment did improve cerebral blood flow at the level of brain MRI imaging evaluation in some cases [25]. However, it may be possible to restore cognitive function after a short period, or even as little as a single dose, assuming the presence of receptors [31]. If, as found in the mouse model of AD, anserine reaches the brain and enhances the function of the cells that make up the microcapillaries in the brain, it could have a rapid effect [29], unlike the long-lasting mechanisms of preventing neuronal death or improving neuronal regeneration. If the drug enhances the function of a group of cells that make up blood vessels, it may have a rapid effect, unlike the long-term mechanism of preventing neuronal death or improving neuronal regeneration.

In our latest human intervention study, imidazole dipeptides, especially anserine, have been reported to possess anti-inflammatory effects on hypochlorite radicals [46]. It is known that, among other radicals, anserine has a potent scavenging effect against hypochlorite radicals produced by MPO. After ingestion of an imidazole dipeptide mixture (carnosine and anserine), carnosine was undetectable in the plasma due to the degradation by plasma carnosinase. On the other hand, it was indeed shown that anserine can remain in plasma, although its half-life is within about one hour [46], confirmed also by a recent LCmsms experiment by Everaert et al. [88]. Once taken up by blood cells such as neutrophils, erythrocytes, or platelets, it was presumed that internally administered anserine had beneficial effects on intracellular homeostasis, metabolic regulation, and ultimately, anti-inflammation. These anti-inflammatory effects in the blood may lead to a protective effect on cerebrovascular sites, leading to the maintenance and improvement of brain function [46].

Regarding the possibility of (2; inside of brain hypothesis), the brain contains carnosine, the basic structure of HCDs, and homocarnosine, a structure in which the beta-alanine moiety in the carnosine molecule is replaced by GABA, which is widely distributed in the brain. However, homocarnosine is present at higher concentrations than carnosine, with homocarnosine at concentrations of about 0.5–1 mM, which can be detected by proton magnetic resonance spectroscopy, while carnosine is not present in the brain to the extent that it can be detected by the same method, suggesting that carnosine may be restricted to the olfactory system [19]. Carnosine is decreased in plasma in AD patients [89], and homocarnosine is decreased in the brain with age.

In the present study, carnosine intake from the normal diet was associated with a higher internal effect of anserine supplementation in improving cognitive function, as plasma carnosine was found to be significantly lower in AD patients [89]. Therefore, it can be inferred that even if carnosine was administered to elderly MCI patients, it would be difficult for the effective dose to reach the cerebral neurovascular system due to its degradation in the plasma. However, if it is not naturally present in the human brain, it may be less susceptible to carnosinase and could reach the brain via the gastrointestinal tract and bloodstream and affect cognitive functions there. Beta-alanine, the enzymatic breakdown product of imidazole dipeptide in the blood, has been shown to remain in the blood for a relatively long time after ingestion of the imidazole dipeptide mixture (containing 250 mg of carnosine and 750 mg of anserine), at a fairly high concentration level. If we assume that this beta-alanine is taken into the brain through the cerebrovascular barrier and that carnosine is newly produced through re-synthesis in the brain tissue, then there is no difference between the effects of anserine and carnosine on the nervous system. The results of the Kyushu University Hisayama-Cho cohort study found that older adults with higher levels of β-alanine in their blood maintained higher cognitive function [90]. To test the possibility of brain re-synthesis, further studies are awaited to verify whether the content in the brain can be altered by non-invasively orally ingested imidazole dipeptide in humans by further increasing the sensitivity of MRS analysis.

Studies reporting the positive effects of carnosine on cognitive function are accumulating, mainly in rodent model studies, where degrading enzymes are not present in the blood. Regarding the mechanism of action of carnosine’s favorable effects on the nervous system in AD, the carnosine-histidine-histamine pathway has been ruled out using histamine receptor antagonists and histidine decarboxylase inhibitors, and amyloid-induced cellular damage is not induced by ROS generation. On the other hand, the use of histamine receptor antagonists and histidine decarboxylase inhibitors has been ruled out, and amyloid-induced cellular damage is not induced by ROS production, making antioxidant activity unlikely [19]. On the other hand, zinc ions can induce amyloid-β aggregation and oligomerization, as well as cytotoxicity, there is a need to address abnormal zinc ion homeostasis in AD. In this regard, carnosine has attracted attention for its chelating activity against metal ions as a mechanism of its neuroprotection [19]. In cultured glial cells, carnosine was found to have a chelating effect on zinc ions [27] and to protect cultured neurons from zinc- and copper-induced toxicity [91]. Zinc ions are important for proper innate immunity but are cytotoxic in excess, especially in cerebral infarction [92]. Indeed, the release of large amounts of zinc, which is abundant in the brain, from synaptic vesicles causes neurotoxicity, and this mechanism has been shown to play a role in neuronal cell death in AD and VaD [34,50,93]. This idea that carnosine exerts a neuroprotective effect in AD through its metal chelating properties suggests that carnosine may act protectively against mechanisms that exacerbate AD pathologies, such as mitochondrial damage, imbalanced neuronal redox status, and abnormal homeostasis of endogenous metals such as copper, iron, and zinc. This is certainly consistent with previous reports showing that carnosine can act protectively against mechanisms that exacerbate the pathology of AD, such as redox status and abnormal homeostasis of endogenous metals such as copper, iron, and zinc. Increased mitochondria-derived ROS production significantly interferes with homeostatic mechanisms that regulate intracellular free zinc ion levels, thereby causing elevated zinc ion concentrations in neurons, leading to a vicious cycle of enhanced and elevated zinc ion-dependent amyloid-β oligomerization and reactive oxygen species formation. In combination with the fact that HCDs can cause a vicious cycle of enhanced and elevated zinc ion-dependent amyloid-β oligomerization and reactive oxygen species formation [27], it is reasonable to speculate that the zinc ion chelating effect of HCDs could be neuroprotective if taken internally and allowed to reach the brain. Although the protective effects of anserine on the central nervous system have not been discussed as much as those of carnosine, possibly that the molecule acting in the brain is not anserine but homocarnosine, which is resynthesized from beta-alanine produced by degradation. Although the increase in the concentration of HCDs in the brain parenchyma after oral administration has not been confirmed, HCDs have a metal chelating effect derived from the imidazole group and may protect the brain in the central nervous system by inhibiting the toxicity of zinc ions released from synaptic vesicles during neuronal excitation in conditions such as AD. The possibility of protection may be another research hypothesis that could be considered.

## 5. Discussion

Within the above-mentioned human study, several neuropsychological tests have been utilized to assess the effect of imidazole dipeptides on the preservation of cognitive function, such as MMSE [94], or WMS-LM Delayed Recall (WMS-DR) [95]. The two tests are mutually independent, and while delayed recall is highly sensitive to memory decline in healthy elderly people [22,23,24,25]. The MMSE test is suitable to evaluate the cognitive function of MCI patients [16,26,46,81]. The CDR is more sensitive for detecting a cognitive decline in MCI [1,6,7,26,82]. The effect of imidazole dipeptides in elderly volunteers with normal cognitive function was detected by the WMS-DR test [22,23,24,25], and the results of MMSE supported the beneficial effect of the imidazole dipeptides on the preservation of cognitive function in individuals with MCI [16,26,46] and patients with probable AD [81]. Besides, MoCA (Montreal Cognitive Assessment) has been also been reported to be sensitive for detecting mild levels of cognitive impairment in elderly individuals [96,97,98], and in a recent study, to evaluate the effect of Matcha green tea formula on the preservation of cognitive function in women [98]. Both MoCa and WMS-DR appeared to be suitable tests to evaluate the effect of nutrients on cognition in elderly individuals whose cognitive function ranged between normality and MCI [99]. Using these neuropsychological tests, we can evaluate the effect of nutrients, including the imidazole dipeptides, for the preservation of cognitive functions.

From a pharmacokinetic point of view, it has been noted that anserine has a short retention time in the blood [46,88]. Daily carnosine intake is likely to support the effect of anserine supplementation [46]. It was very difficult to detect the carnosine in the plasma after the supplementation [46]. A recent result of metabolomics analysis in the elderly has yielded some interesting research findings. It has been reported that the plasma levels of carnosine and acetyl-carnosine are significantly decreased in the elderly compared to the young [100]. In patients with AD, the plasma concentration of carnosine is decreased, while the concentration of anserine is increased [89]. The relationship between these changes in blood concentrations under normal conditions and after ingestion must await further study, but is likely to provide some hints for future research and development.

Imidazole dipeptides such as anserine and carnosine are also expected to be taken up by blood cells. In this process, the transporter PEPT2 is thought to be utilized [20]. It has been suggested that imidazole dipeptides may be taken up by red blood cells, platelets, and even neutrophils [46], and after being taken up into cells, they may be taken up into each vesicle in the cell via the endoplasmic reticulum-type transporters PHPT1/2, contributing to homeostasis in the microenvironment. It has been reported that imidazole dipeptides are incorporated into the mitochondrial membrane [19,27]. After being taken up by these cells, it is presumed to play a role in the maintenance of intracellular homeostasis, metabolic regulation, and ultimately anti-inflammatory effects. These anti-inflammatory effects in the blood may lead to protective effects in cerebral vascular sites [29], leading to the maintenance and improvement of brain function [22,25], but further analysis is still needed.

Carnosine was initially assumed to be effective in removing senile plaques due to its high affinity for amyloid peptides [55] and to have ameliorating effects on dementia [27]. Studies on model mice showed that long-term administration of carnosine affected the suppression of the accumulation of senile plaques [27], and it also had protective and anti-inflammatory effects on brain cells [28]. A similar low-molecular-weight prodrug is a homotaurine [101]. Homotaurine was also shown to eliminate senile plaques and to have pharmacokinetics that could pass through the BBB, and a large-scale phase 3 study was conducted in humans. However, the overall effect could not be confirmed [102], and it is now distributed as a supplement. Subsequent stratified analysis showed significant effects, including improvement in hippocampal atrophy [103], in those with risk factors for AD (ApoE4/E4) [104], and a Phase 3 study will be conducted using valine modified valinyl-homotaurine (ALZ801), which was more effective in terms of side effects and pharmacokinetics [105,106]. Imidazole dipeptides such as anserine and carnosine have a similar history of developmental research but have not yet been fully investigated for modification [107,108]. In this sense, we are waiting for the clarification of the mechanism of action of imidazole dipeptides, which may have an aversive effect on brain aging and the development of dementia.

## 6. Conclusions

Imidazole dipeptides, integral components of vertebrate muscle tissues, have anti-inflammatory and antioxidant properties. Experimental studies in animal models and controlled clinical trials in elderly individuals indicate that these compounds also have many protective effects on brain aging. Recently, administration of the imidazole dipeptide anserine for 12 weeks improved cognitive function in elders with MCI, suggesting a preventive effect against dementia. While the mechanism of action of imidazole dipeptides on neuroprotection remains largely unknown, the evidence thus far points to a beneficial role in age-related cognitive dysfunction.

## Figures and Tables

**Table 1:** Main characteristics of the imidazole dipeptide studies in Alzheimer’s disease (AD) model mouse.

Authors and Year	Type of AD Model Mouse	Start of Treatment, Trial Duration	Sex, Group & Sample Size	Treatment (Doses)	Outcomes of Interest
Corona et al., 2011 [27]	3xTg-AD	1 month, 12 months	MF, control (11), 3× Tg-AD (13), 3× Tg-AD + Carnosine (9)	10 mg of carnosine per day (400 mg/kg/day)	Morris water maze, Abeta-load, Mitochondria function, Zinc chelating
Herculano et al., 2013 [28]	App/psen + High Fat Diet	4 month, 6 weeks	MF, WT (11); AD (13); AD+HFD (13); AD+HFD + Carnosine (11)	5 mg of carnosine per day (200 mg/kg/day)	Contextual fear conditioning, Abeta-load, Neuroinflammation
Kaneko et al., 2017 [29]	App/psen	18 month, 8 weeks	MF, WT (10); WT + Ans (11); AD (10); AD + Ans (10)	10 mg of anserine per day (400 mg/kg/day)	Morris water maze, Abeta-load, Neuroinflammation, Neurovascular damage

**Table 2:** Main characteristics of the imidazole dipeptide studies for elders.

Authors and Year	Study, Country	Trial Duration, Sample Size (Act: Pla)	Gender, Age (Mean ± SD) Group;	Subjects; Cognitive Function	Imidazole Dipeptide; (Doses)	Outcomes of Interest
Szcześniak et al., 2014 [16]	Double-blind, Placebo-controlled, Randomized controlled trial (DbPcRCT), Poland	13 weeks 51 (26: 25)	MF, 81.0 ± 7.0 y Active; 80.5 ± 7.5 y Placebo	Residents in a Nursing home; MMSE, >15	1 g of anserine/ carnosine (2:1 ratio); once a day	Cognitive function (MMSE, STMS), depression (GDS)
Katakura et al., 2017 [24]	DbPcRCT, JAPAN	12 weeks 60 (30: 30)	MF, 60.4 ± 2.1 y Active; 65.3 ± 1.6 y Placebo	Community-dwelling Healthy volunteers MMSE ≥ 24	0.5 g of anserine/ carnosine (3:1 ratio); twice a day	Cognitive function (MMSE), Alzheimer’s disease (ADAS), memory (WMS-LM1, WMS-LM2), depression (BDI)
Ding et al., 2018 [25]	DbPcRCT, JAPAN	12 months (extension of Hisatsune et al., 2016) [23] 68 (31: 37)	MF, 71.3 ± 4.8y Active; 71.8 ± 4.8y Placebo	Community-dwelling Healthy elderly volunteers MMSE > 24	0.5 g of anserine/ carnosine (3:1 ratio); twice a day	Memory (WMS-LM1, WMS-LM2), Alzheimer’s disease (ADAS) depressive symptoms (BDI) MRI (ASL, DTI)
Hisatsune et al., 2016 [23]	DbPcRCT, JAPAN	(1) 6 months 84 (42: 42) (2) 12 weeks 39 (19: 20) (part of Katakura et al., 2017) [24]	(1) MF, 69.4 ± 5.9 y Active; 70.4 ± 5.7 y Placebo (2) MF, 67.8 ± 5.6 y Intervention, 70.6 ± 5.1 y Control	Community-dwelling Healthy elderly volunteers MMSE ≥ 24	0.5 g of anserine/ carnosine (3:1 ratio); twice a day	Memory (WMS-LM1, WMS-LM2), Alzheimer’s disease (ADAS) depressive symptoms (BDI) MRI(ASL)
Rokicki et al., 2015 [22]	DbPcRCT, JAPAN	12 weeks 31 (14: 17) (part of Katakura et al., 2017) [24]	MF, 61.4 y Active; 66.5 y Placebo	Community-dwelling Healthy elderly volunteers MMSE ≥ 24	0.5 g of anserine/carnosine (3:1 ratio); twice a day	Memory (WMS-LM1, WMS-LM2), (ADAS) depressive symptoms (BDI) MRI(fMRI)
Masuoka et al., 2019 [26]	DbPcRCT, JAPAN	12 weeks 50 (25: 25)	MF, 72.9 ± 8.8 y Active; 73.6 ± 6.1 y Placebo	Outpatients with MCI, MMSE ≥ 24	375 mg anserine and 125 mg carnosine; twice a day	Cognitive function (MMSE), Alzheimer’s disease (ADAS), dementia (CDR), memory (WMS), depressive symptoms (GDS)
Cornelli et al., 2010 [81]	DbPcRCT, USA	6 months 48 (23: 25)	MF, 75.0 ± 4.2 y Active; 74.0 ± 4.9 y Placebo	Patients with diagnosis of probable AD, MMSE score > 21	Formula F (100 mg carnosine and anti-oxidant*); once per day	Cognitive function (MMSE)
Masuoka et al., in press [46]	DbPcRCT, JAPAN	12 weeks 30 (15; 15)	MF, 74.5 ± 4.6 y Active; 72.0 ± 5.2 y Placebo	Community-dwelling elderly people with MCI, MoCA ≤ 25	250 mg anserine; twice a day	Cognitive function (MMSE), Alzheimer’s disease (ADAS); Inflamma-tion (Plasma CRP)

Abbreviations: ADAS (Alzheimer’s Disease Assessment Scale); BDI (Beck Depression Inventory); CDR (Clinical Dementia Rating); CRP (C-Reactive Protein); DbPcRCT (Double-blind, Placebo-controlled, Randomized controlled trial); F (female); DTI (Diffusion Tensor Imaging); GDS (Geriatric Depression Scale); M (male); MCI (mild cognitive impairment); MMSE (Mini Mental State Examination); MoCA (Montreal Cognitive Assessment); STMS (Short Test of Mental Status); WMS-LM (Wechsler Memory Scale Logical Memory); y (years).* vitamins B, vitamin C and E, coenzyme Q10, beta-carotene, selenium, l-cysteine, Ginko biloba.
